# Role of dopamine D2 receptors in optimizing choice strategy in a dynamic and uncertain environment

**DOI:** 10.3389/fnbeh.2014.00368

**Published:** 2014-10-28

**Authors:** Shinae Kwak, Namjung Huh, Ji-Seon Seo, Jung-Eun Lee, Pyung-Lim Han, Min W. Jung

**Affiliations:** ^1^Center for Synaptic Brain Dysfunctions, Institute for Basic ScienceDaejeon, Korea; ^2^Neuroscience Laboratory, Institute for Medical Sciences, Ajou University School of MedicineSuwon, Korea; ^3^Neuroscience Graduate Program, Ajou University School of MedicineSuwon, Korea; ^4^Department of Brain and Cognitive Sciences, Ewha Womans UniversitySeoul, Korea; ^5^Department of Biological Sciences, Korea Advanced Institute of Science and TechnologyDaejeon, Korea

**Keywords:** D1 receptor, D2 receptor, reversal, dynamic foraging task, mouse, reinforcement learning

## Abstract

In order to investigate roles of dopamine receptor subtypes in reward-based learning, we examined choice behavior of dopamine D1 and D2 receptor-knockout (D1R-KO and D2R-KO, respectively) mice in an instrumental learning task with progressively increasing reversal frequency and a dynamic two-armed bandit task. Performance of D2R-KO mice was progressively impaired in the former as the frequency of reversal increased and profoundly impaired in the latter even with prolonged training, whereas D1R-KO mice showed relatively minor performance deficits. Choice behavior in the dynamic two-armed bandit task was well explained by a hybrid model including win-stay-lose-switch and reinforcement learning terms. A model-based analysis revealed increased win-stay, but impaired value updating and decreased value-dependent action selection in D2R-KO mice, which were detrimental to maximizing rewards in the dynamic two-armed bandit task. These results suggest an important role of dopamine D2 receptors in learning from past choice outcomes for rapid adjustment of choice behavior in a dynamic and uncertain environment.

## Introduction

There has been a large progress in understanding roles of dopamine in reward processing over the last two decades. In particular, the finding that midbrain dopamine neurons signal the difference between actual and expected rewards (Schultz et al., 1997) led to the proposal that midbrain dopamine neurons signal reward prediction error (RPE; the difference between actual and predicted rewards) as postulated by the reinforcement learning (RL) theory (Sutton and Barto, 1998). In RL, a decision maker assigns a value funciton (a sum of expected future rewards) to each available action and makes choices based on a set of value functions in order to maximize a long-term sum of rewards. In turn, value functions are updated according to the difference between actual and predicted rewards (i.e., RPE). A large body of subsequent studies employing the RL theory have yielded results that further support the role of dopamine in updating value functions according to RPE (Daw and Doya, 2006; Dayan and Niv, 2008; Kable and Glimcher, 2009; Niv and Montague, 2009; Lee et al., 2012). This line of research emphasizes an essential role of dopamine in learning to choose optimally for maximizing rewards. However, dopamine-deficient animals can learn to choose more rewarding targets (Berridge, 2007) and some dopamine neurons signal stimulus salience rather than RPE (Brischoux et al., 2009; Matsumoto and Hikosaka, 2009; Wang and Tsien, 2011), which led to the proposal that the primary role of dopamine might be in forming incentive salience rather than learning to choose more rewarding targets. In addition, dopamine involvement in another aspect of RL, namely controlling exploration-exploitation trade-off, has been proposed. In a dynamic environment, it is advantageous for a decision maker to choose an action with a low value function from time to time (exploration) rather than to exclusively choose an action with the highest value function (exploitation) in order to keep track of dynamically changing value functions. Previous theoretical and empirical studies have suggested involvement of dopamine in controlling exploratory vs. exploitive choices (Frank et al., 2009; Beeler et al., 2010; Humphries et al., 2012). As such, the extent and nature of dopamine roles in RL are still under debate.

We investigated this matter using mice while manipulating stability and certainty of action-reward contingency. Given that the core concept of RL is to discover optimal choice strategy in a dynamic and uncertain environment (Sutton and Barto, 1998), it would be desirable to employ a behavioral task that emulates dynamicity and uncertainty in action-reward contingency in investigating the role of dopamine in RL. However, unlike in human studies (e.g., Frank et al., 2004, 2007, 2009; Pessiglione et al., 2006; Klein et al., 2007; Rutledge et al., 2009), animal studies seldom tested effects of dopamine manipulation in a behavioral task wherein action-reward contingency was uncertain and dynamically varied in the context of value-based decision making, which undermines an important advantage of using animal over human subjects (i.e., comprehensive and complete manipulation of dopamine). In the present study, to manipulate stability and certainty of action-reward contingency, we tested mice in a simple instrumental learning task with the frequency of reversal progressively increased (i.e., dynamicity of action-reward contingency was gradually increased) and a dynamic two-armed bandit (TAB) task in which binary choices were associated with different reward probabilities (i.e., uncertainty was added). In addition, we examined effects of manipulating dopamine receptor subtypes rather than dopamine itself. Specifically, we examined choice behavior of dopamine D1 and D2 receptor knock-out (D1R-KO and D2R-KO, respectively) mice. Dopamine D1R and D2R are major subtypes among five subtypes of dopamine receptors, and their anatomical distributions and functional roles are distinct (Hurley and Jenner, 2006; Kreitzer and Malenka, 2008). These features call for separate manipulations of D1R and D2R to fully capture the extent of dopamine functions. For these reasons, we examined choice behavior of D1R-KO and D2R-KO mice while manipulating stability and certainty of action-reward contingency.

## Materials and methods

### Subjects

D1R-KO and D2R-KO mice were described previously (Drago et al., 1994; Kelly et al., 1997) and were purchased from JAX lab (Bar Harbor, Maine, USA). They were bred to C57BL/6J mice for more than 10 generations in our lab. All D2R^−/−^ mice and their wild type (WT) were obtained by crossing D2R^+/−^ and D2R^+/−^, and had C57BL/6J genetic background. Few D1R^−/−^ mice with C57BL/6J genetic background survived to adult stage. To overcome this problem, D1R^+/−^ were backcrossed with 129S1/SvlmJ and their F1 progenies were crossed with 129S1/SvlmJ again. Crossing D1R^+/−^ and D1R^+/−^ of F2 progenies gave rise to D1R^−/−^ mice, which were, together with WT littermates as control, used in this study. Therefore, all D1R^−/−^ mice and their WT control had C57BL/6J-129S1/SvlmJ hybrid genetic background. For genotype analysis, the following primer sets were used: 5′-AAA GTT CCT TTA AGA TGT CCT-3′ and 5′-TGG TGG CTG GAA AAC ATC AGA-3′ for D1R (350 bp); 5′-TGT GAC TGC AAC ATC CCA CC-3′ and 5′-GCG GAA CTC AAT GTT GAA GG-3′ for D2R (105 bp); 5′-CTT GGG TGG AGA GGC TAT TC-3′ and 5′-AGG TGA GAT GAC AGG AGA TC-3′ for the KO state (neo; 280 bp) in both KO cases. The experimental protocol was approved by the Institutional Animal Care and Use Committees of Ajou University School of Medicine, Ewha Womans University, and Korea Advanced Institute of Science and Technology.

### Apparatus

**Figure 1:**
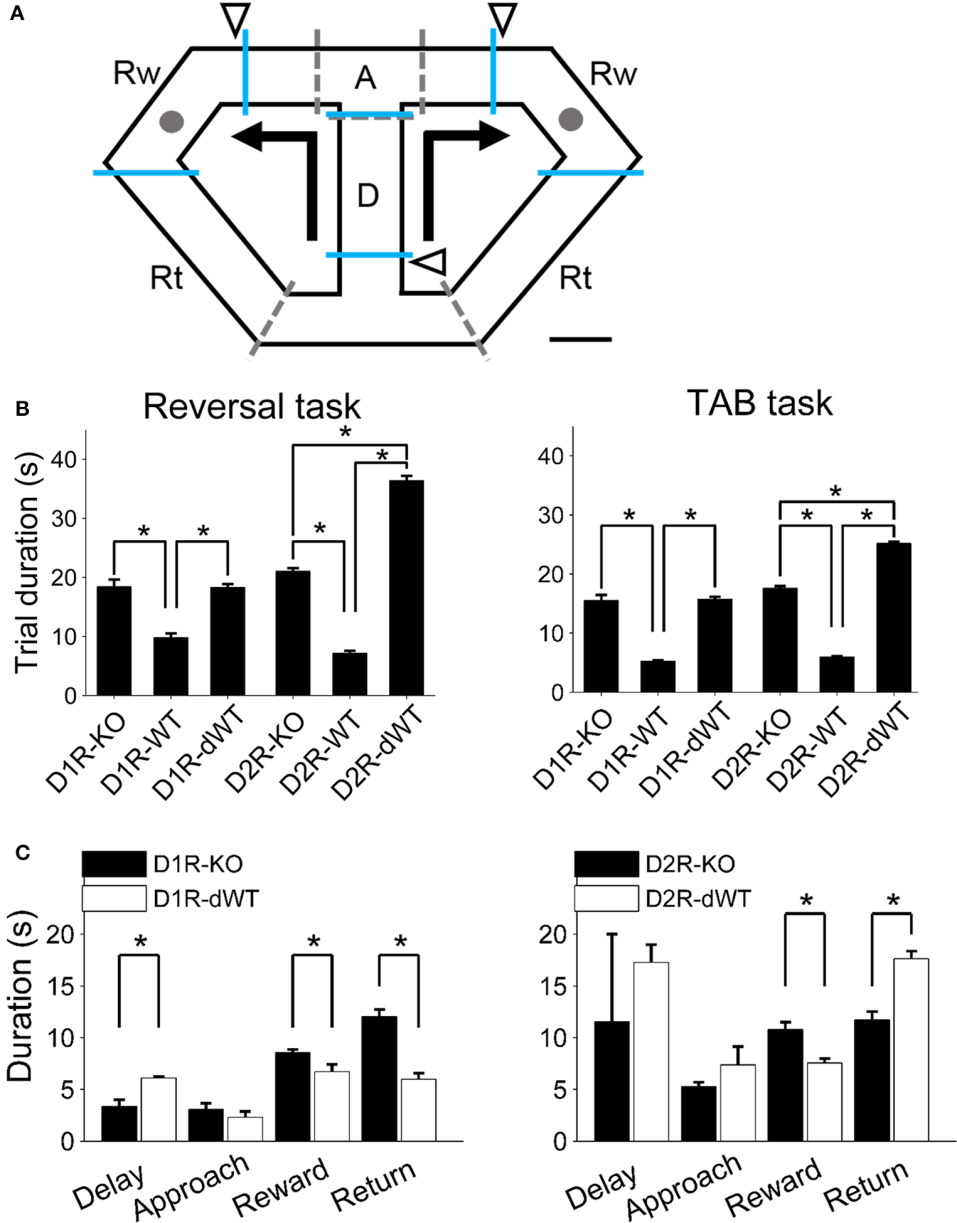
**Behavioral task and experimental groups. (A)** Left, A schematic diagram of the modified T-maze used in the present study. Mice were allowed to choose between two target locations (gray circles) that delivered water reward. Arrows indicate movement directions that were controlled by opening and closing of 5 sliding doors (indicated by dashed lines). Triangles indicate locations of photobeam sensors. The maze was divided into four sections (blue lines; D, delay; A, approach to goal; Rw, reward; Rt, return to central stem). Scale bar, 6 cm. **(B)** Mean trial durations (±s.e.m.) during the reversal and TAB tasks are shown for each experimental group. There were three experimental groups for each receptor subtype [KO, WT, and WT with delay (dWT)]. Delay was imposed to dWT animals on the central stem in order to match their trial durations to those of KO mice. Trial duration was not significantly different between D1R-KO and D1R-dWT mice (reversal task, *p* = 0.913, TAB task, *p* = 0.767; One-Way ANOVA followed by Bonferroni *post-hoc* tests) but significantly longer for D2R-dWT than D2R-KO mice (reversal task, *p* = 2.3 × 10^−45^, TAB task, *p* = 1.4 × 10^−48^). Asterisks, significant differences (*p* < 0.05). Six D2R-WT and five D2R-KO mice were excluded from calculating trial durations in the TAB task because trial durations were not measured for them. **(C)** Mean durations of the delay, approach, reward and return phases are shown for D1R-KO, D1R-dWT, D2R-KO, and D2R-dWT mice for the TAB task. Asterisks, significant differences (*t*-test, *p* < 0.05).

The animals were trained on a modified T-maze (Figure 1A) that was made of black acrylic (overall dimension, 55 × 30 cm; width of track: 6 cm with 10-cm high walls along the entire track). It was elevated 80 cm from the floor and covered with a transparent acrylic lid. Water (10~15 μl; same amount for a given animal) was delivered by briefly opening a solenoid valve at the upper left and upper right corners. The maze contained five sliding doors to guide movement direction and to impose delay on the central stem for some animals (delay-imposed WT animals; see below). Navigation of the animal was monitored by three sets of photobeam sensors that signaled the animal's arrival at the goals and return to the start arm. Monitoring of animal behavior and water delivery were automatically controlled by a personal computer using LabView software (National Instruments, TX, USA).

### Behavioral tasks

The animals were tested in a reversal task and a dynamic TAB task. In both tasks, they were placed on the central stem of the maze and allowed to choose freely between two goals that delivered water reward. They were required to come back to the central stem via the lateral alleys. Sliding doors were opened or closed when appropriate to guide navigation of the animals (Figure 1A).

#### Reversal learning task

One goal delivered water with 100% probability and the other with 0%. Locations of the correct and incorrect goals were reversed initially across sessions, and then within a session. For this, the animals went through five stages of testing as follows: stage 1, training without reversal (the location of correct goal was counterbalanced across animals; 45–60 daily trials), 3 d; stage 2, reversal of target location at the beginning of the first session and training without further reversal, 4 d (60 daily trials); stage 3, reversal of target location at the beginning of each session, 4 d (60 daily trials); stage 4, one episode of target location reversal in the middle of each session, 4 d (60 daily trials with reversal at trial #31); stage 5, two episodes of target location reversal in the middle of each session, 4 d (90 daily trials with reversal at trials #31 and 61). For stages 4 and 5, the initial location of the correct target was randomly determined for each session except on day 1 (reversal of target location from the previous day).

#### Dynamic TAB task

Two goals delivered water with different probabilities in the dynamic TAB task (Kim et al., 2009; Sul et al., 2010). Reward probability of a goal was constant within a block of trials but was changed across blocks without any sensory cues. The mice therefore had to detect changes in relative reward probabilities by trial and error. The number of trials in each block was between 35 and 55. The order of block reward probabilities in a given session was determined randomly with the constraint that the option with the higher reward probability always changed its location at the beginning of a new block. All animal groups went through at least three stages of training (stages 1–3) that employed different arming probabilities as follows: stage 1, two blocks, 0.84 vs. 0.12; stage 2, three blocks, 0.84 vs. 0.14; stage 3, three blocks, 0.80 vs. 0.16. D2R animal groups went through two additional stages of training (stages 4–5) with arming probabilities as follows: stage 4, three blocks, 0.72 vs. 0.24; stage 5, four blocks, 0.72 vs. 0.12 and 0.63 vs. 0.21. This was to test whether poor performance of D2R-KO animals (see below) could be overcome by prolonged training. The animals were trained for 10 d in stages 1–4, and for 60 d in stage 5. However, D2R-KO animals were further trained for 10 additional days in each of stages 1–4 (total 20 d of training in each stage). Again, this was to test whether prolonged training can enhance performance of D2R-KO mice.

All animals initially went through 2–3 days of acclimation to the maze and a shaping period. Of the animals tested in both tasks, the sequence of the tasks was counterbalanced across the animals (18 and 13 were tested first in the reversal and dynamic forging task, respectively), and they were kept in their home cages for 2–3 weeks between the two phases of training to minimize interference between the two tasks.

### Experimental groups

D1R-KO and D2R-KO mice were compared with their respective WT littermates (C57BL/6J-129S1/SvlmJ and C57BL/6J, respectively). Also, because D1R-KO and D2R-KO mice were slower in performing the behavioral tasks, we imposed delay on the central stem for separate groups of WT littermates to match their trial durations to those of mutant mice. Therefore, there were three experimental groups [WT, WT with delay (dWT) and KO] for each receptor type. A fixed length of delay (the difference in mean trial duration between D2R-KO and D2R-WT; 15 s in the reversal task and 10 s in the TAB task) was imposed to D2R-dWT mice, which resulted in longer mean trial durations for D2R-dWT than D2R-KO mice. For D1R-dWT mice, to avoid this problem, delay durations were adjusted in blocks of 10 trials based on mean delay durations of D1R-KO and D1R-dWT mice up to that time point so that the final mean delay duration was similar between the two animal groups for a given training stage (Figure 1B). We divided the maze into four sections (delay, approach, reward and return sections; Figure 1A) and measured time the animals spent in each section during the TAB task to compare response patterns of the KO and dWT mice. For this, for each of D1R-KO, D1R-dWT, D2R-KO, and D2R-dWT animal groups, 1000 trials were evenly divided to each animal and each training day of stages 1–3, and then the resulting number of trials for a given animal for a given training day was randomly selected for analysis. D1R-dWT mice spent less time at the reward site and in coming back from a goal site to the central stem than D1R-KO mice, as expected. D2R-dWT mice also spent less time at the reward site, but were slower in coming back from a goal site to the central stem than D2R-KO mice (Figure 1C). This might be because D2R-dWT mice were trapped in the central section (“D” in Figure 1A) for 15 (reversal task) or 10 s (TAB task) in each trial (they might be reluctant to enter the central section), whereas the duration of delay was variable across blocks of 10 trials for D1R-dWT mice. Other than these differences between KO and dWT mice, we were unable to identify unusual behavior of KO mice on the maze.

Note that different trial durations of D2R-KO and D2R-dWT mice did not affect the conclusions because the performance of D2R-KO mice was impaired relative to D2R-dWT mice which had longer, rather than shorter, trial durations. Also, similar results were obtained when the animal's performance was analyzed with a logistic regression analysis that took trial-by-trial variations in trial duration into account, and when model-based analysis was performed after matching trial durations between D2R-KO and D2R-dWT mice groups by excluding some behavioral sessions (see below). Of a total of 48 animals, the majority (*n* = 31, 64.6%) were tested in both behavioral tasks, seven (14.6%) were tested only in the reversal task, and ten (20.8%) were tested only in the TAB task. Nine D1R-WT, six D1R-dWT, four D1R-KO, five D2R-WT, five D2R-dWT, and nine D2R-KO mice were tested in the reversal task, and nine D1R-WT, six D1R-dWT, three D1R-KO, nine D2R-WT, five D2R-dWT, and nine D2R-KO mice were tested in the dynamic TAB task.

### Analysis

#### Logistic regression analysis

Two different logistic regression analyses were used. The first logistic regression analysis was to examine effects of D1R-KO and D2R-KO on the animal's performance controlling for trial-by-trial variations in trial duration. For this, we related the animal's choice with the animal type (KO vs. WT) and trial duration as the following:

$$\log\left(\frac{p_{High}(i)}{p_{Low}(i)}\right)=a_{G}X_{G}+a_{T}T(i)+a_{0},$$

where *p*_*High*_ (*i*) (or *p*_*Low*_ (*i*)) is the probability of selecting the direction with a higher (or lower) reward probability in the *i*-th trial, and *X*_*G*_ is a dummy variable representing the animal type (KO vs. WT) and *T*(*i*) is the trial duration in the *i*-th trial.

The second regression analysis was to examine how the animal's choices and their outcomes in the past 10 trials influenced the animal's choice in the current trial in the TAB task. For this, the following logistic regression analysis was performed (Lau and Glimcher, 2005; Huh et al., 2009; Kim et al., 2009):

$$\begin{array}{l} \log\left(\frac{p_{L}(i)}{p_{R}(i)}\right)=\sum_{j\;=\;1}^{10}\gamma_{j}^{r}\left(R_{L}(i-j)-R_{R}(i-j)\right)\\ \;+\sum_{j\;=\;1}^{10}\gamma_{j}^{c}\left(C_{L}(i-j)-C_{R}(i-j)\right)+\gamma_{0}, \end{array}$$

where *p*_*L*_ (*i*) (or *p*_*R*_ (*i*)) is the probability of selecting the left (or right) goal in the *i*-th trial. The variables *R*_*L*_ (*i*) (or *R*_*R*_ (*i*)) and *C*_*L*_ (*i*) (or *C*_*R*_ (*i*)) are reward delivery at the left (or right) goal (0 or 1) and the left (or right) goal choice (0 or 1) in the *i*-th trial, respectively. The coefficients γ^*r*^_*j*_ and γ^*c*^_*j*_ denote the effect of past rewards and choices, respectively, and γ_0_ is a bias term. The regression model was applied separately for each animal using the entire choice data during the TAB task (D1R, stages 1–3; D2R, stages 1–5).

#### Models of behavior

In order to obtain insights on psychological/neural processes underlying the animal's choice behavior, we tested how well different models can account for the animal's choice behavior during the TAB task. The full model contained simple RL, win-stay-lose-switch, and uncertainty-based exploration terms along with choice bias, and one or more of these terms were left out in reduced models. In the full model, win-stay-lose-switch and uncertainty-based exploration terms for the chosen action “*a*” and unchosen action “*b*” (left or right goal choice) were determined as the following:

$$\begin{array}{l} U_{a}(t)=S_{a}(t)+\rho \mu_{a}(t)+\varepsilon \sigma_{a}(t),\\ U_{b}(t)=S_{b}(t)+\rho \mu_{b}(t)+\varepsilon \sigma_{b}(t), \end{array}$$

where *S*_*a*_(*t*) and *S*_*b*_(*t*) are win-stay (WS) and lose-switch (LS) terms, respectively [*S*_*a*_(*t*) = *S*_*reward*_ and *S*_*b*_(*t*) = 0 if rewarded in the previous trial and *S*_*a*_(*t*) = 0 and *S*_*b*_(*t*) = *S*_0_ otherwise], and μ_*a*_(*t*) and σ_*a*_(*t*) determine uncertainty-based exploration in the *t*-th trial. Contributions of the factors for the uncertainty-based exploration were quantified by the free parameters ρ and ε. μ_*a*_(*t*) is the mean reward value computed from the reward structure experienced in the past trials and σ_*a*_(*t*) is the SD of the distribution of the estimated reward structure. For the estimation of the mean expected values from the experienced reward history, we used the Kalman filter (Kruschke, 2008; Frank et al., 2009) and μ_*a*_(*t*) was assumed to follow the normal distribution *N*(μ, σ^2^). The values μ_*a*_(*t*) and σ_*a*_(*t*) were updated for the chosen action “*a*” as the following:

$$\begin{array}{l} \mu_{a}(t+1)=\mu_{a}(t)+k_{a}(t)\left\{R(t)-\mu_{a}(t)\right\},\\ \;k_{a}(t)=\frac{\sigma_{a}{\left(t\right)}^{2}}{\sigma_{a}{\left(t\right)}^{2}+\sigma_{reward}{\left(t\right)}^{2}}, \end{array}$$

where *k*_*a*_(*t*) is the Kalman gain and σ_*reward*_(*t*) is the SD of the actual rewards taken by the mice. σ_*a*_(*t*) was computed as the following:

$$\sigma_{a}(t+1)=\sigma_{a}(t)\left\{1-k_{a}(t)\right\}.$$

For the unchosen action “*b*,” the values μ_*b*_(*t*) and σ_*b*_(*t*) were unchanged.

For the RL term, a Q-learning model (Sutton and Barto, 1998) was used. Briefly, action values were updated based on RPE in each trial as the following:

$$\begin{array}{l} \text{if }choice=left,\;RPE=R(t)-Q_{L}(t),\\ \;Q_{L}(t+1)=Q_{L}(t)+\alpha \cdot RPE,\\ \text{ ​ }Q_{R}(t+1)=Q_{R}(t),\\ \text{if }choice=right,\;RPE=R(t)-Q_{R}(t),\\ \;Q_{R}(t+1)=Q_{R}(t)+\alpha \cdot RPE,\\ \text{ ​​ }Q_{L}(t+1)=Q_{L}(t), \end{array}$$

where α is the learning rate, *Q*_*L*_(*t*) and *Q*_*R*_(*t*) are action values for leftward and rightward choices, respectively, and *R*(*t*) is the reward in the *t*-th trial (1 if rewarded and 0 otherwise). The learning rate was different depending on the sign of RPE as the following: α = α_*pos*_ if *RPE* >0, and α = α_*neg*_ otherwise.

Choices were made according the softmax action selection rule as the following:

$$p_{L}(t)=\frac{1}{1+\exp(-\beta (Q_{L}(t)-Q_{R}(t))-(U_{L}(t)-U_{R}(t)))},$$

where *p*_*L*_(*t*) is the probability for selecting the left goal and β is the inverse temperature that determines the degree of randomness in action selection. Model parameters were estimated separately for each animal based on the entire choice data (D1R, stages 1–3; D2R, stages 1–5) using fminsearch function of MATLAB (Mathwork Inc.).

### Statistical tests

Two-Way repeated measure ANOVA was applied separately to the behavioral data obtained from D1R and D2R animal groups to examine effects of experimental groups (KO, WT, and dWT) and training days on the animal's behavioral performance. For the stable phases of the TAB task (stages 2–5), behavioral performance data were collapsed across stages 2–3 (D1R and D2R animal groups) and stages 4–5 (only D2R animal groups) and analyzed with One-Way ANOVA. ANOVA was followed by Bonferroni *post-hoc* tests (SPSS 20). Model parameters of KO and dWT animal groups were compared with Wilcoxon rank-sum tests (two-tailed). Statistical significance of the regression coefficients was tested with *t*-tests (two-tailed). A *p*-value < 0.05 was used as a criterion for significant difference. The data are expressed as mean ± s.e.m.

## Results

### Performance in the reversal task

**Figure 2:**
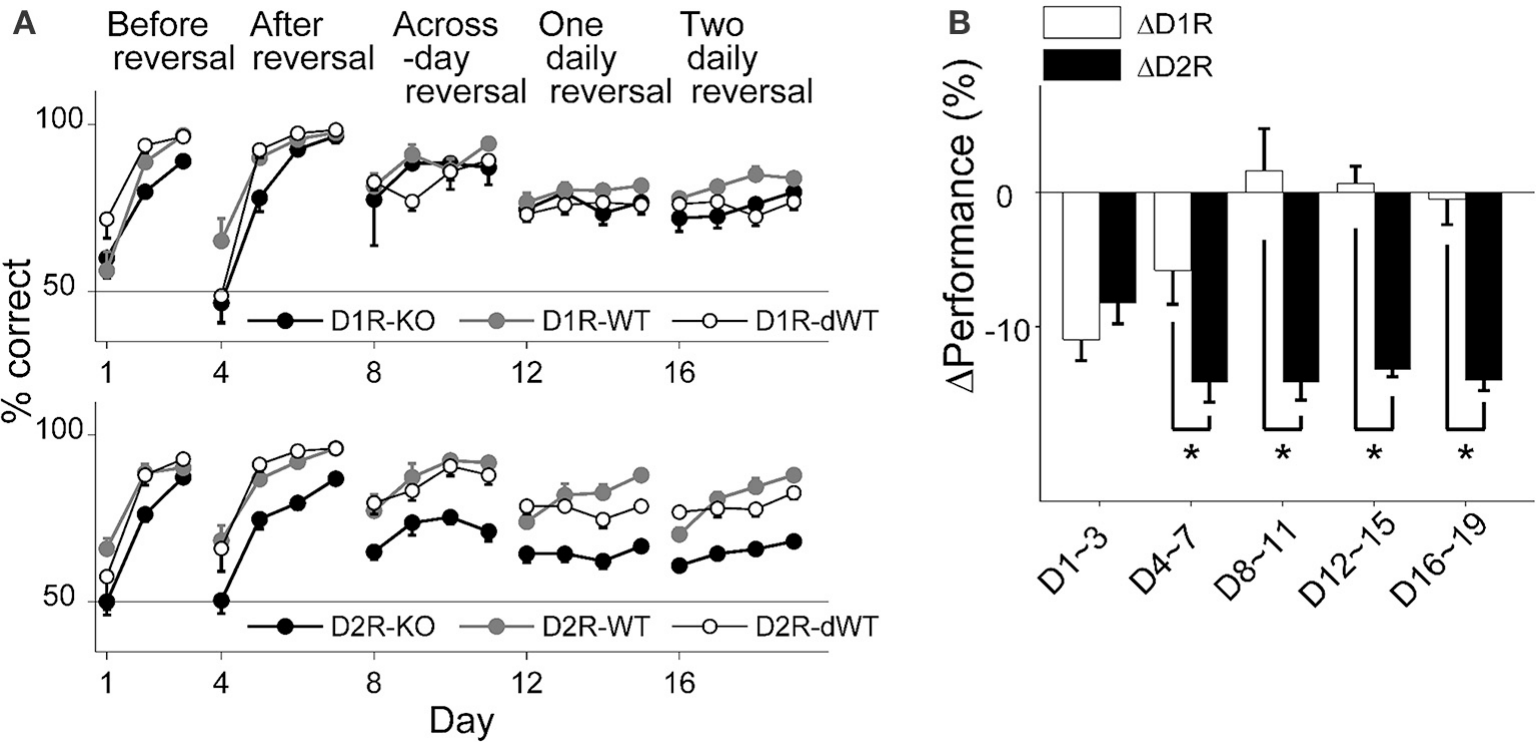
**Behavioral performance in the reversal task. (A)** Behavioral performance (% correct choice; mean ± s.e.m. across animals) is shown for each daily session for each experimental group. **(B)** Relative performance (performance difference between KO and dWT groups; mean ± s.e.m. across daily sessions) is shown for each training stage. Asterisks, significant differences (*t*-test, *p* < 0.05).

All animal groups learned to choose the correct goal over the initial 3 days of training (stage 1; Two-Way repeated measure ANOVA, main effect of training day, D1R, *p* = 1.0 × 10^−6^; D2R, *p* = 4.0 × 10^−6^), during which the location of the correct goal was fixed, so that the animal's performance (% correct choice) on the third day of training was >80% in all animal groups (Figure 2A). However, D2R-KO mice were slower in improving performance compared to the other animal groups (main effect of animal group, *p* = 0.004; Bonferroni *post-hoc* test, D2R-KO vs. D2R-WT, *p* = 0.007; D2R-KO vs. D2R-dWT, *p* = 0.035). D1R-KO mice also showed a trend for lower performance compared to D1R-dWT mice (main effect of animal group, *p* = 0.045; D1R-KO vs. D1R-dWT, *p* = 0.054). Upon the reversal of the correct goal location, all animals learned to choose the new target location (stage 2; days 4–7; main effect of training day, D1R, *p* = 1.0 × 10^−6^; D2R, *p* = 1.0 × 10^−7^) so that the animal's performance on the fourth day (day 7) of training was >80% (Figure 2A). However, performance of D2R-KO mice was lower (main effect of animal group, *p* = 8.0 × 10^−6^) relative to those of D2R-WT (*p* = 9.3 × 10^−5^) and D2R-dWT (*p* = 3.1 × 10^−5^) mice. D1R-KO mice showed lower performance than the other animals groups only on day 5 (main effect of animal group, *p* = 0.160; group × day interaction, *p* = 0.012; day 5, D1R-KO vs. D1R-WT, *p* = 0.026; D1R-KO vs. D1R-dWT, *p* = 0.013).

D1R-KO mice showed relatively intact performance during subsequent stages of reversal training (Figure 2A). No significant performance variation was found within D1R animal groups during across-session reversal (stage 3; correct goal location changed at the beginning of each session, days 8–11; main effect of animal group, *p* = 0.590) or one-daily reversal (stage 4; correct goal location changed once during each session; days 12–15; *p* = 0.233). The only significant difference was found between D1R-WT and D1R-dWT animals on day 18 (*p* = 0.007). By contrast, performance of D2R-KO mice was significantly impaired in all subsequent stages of reversal training (main effect of animal group, *p*-values < 9.0 × 10^−5^) compared to D2R-WT (*p*-values < 3.0 × 10^−4^) and D2R-dWT (*p*-values < 2.0 × 10^−4^; Figure 2A).

To further control for trial-by-trial variations in trial duration, we examined effects of D1R-KO and D2R-KO on the animal's performance using a logistic linear regression analysis that included trial-by-trial duration as an explanatory variable (see Materials and Methods). This analysis indicated significant effect of D1R-KO on the animal's performance during stage 1 (*t*-test, *p* = 7.8 × 10^−10^), 4 (*p* = 7.8 × 10^−9^) and 5 (*p* = 6.3 × 10^−15^), but not during stages 2 and 3 (*p* = 0.921 and 0.382, respectively), and significant effect of D2R-KO in all training stages (*p*-values < 3.0 × 10^−5^). For direct comparison between D1R-KO and D2R-KO animals, we compared relative performance, which is the difference in performance (% correct choice) between KO and dWT groups (ΔD1R and ΔD2R). ΔD2R was significantly lower than ΔD1R in all training stages except the first (*t*-test, stages 1–5, *p* = 0.369, 0.048, 0.007, 1.2 × 10^−4^, and 0.001, respectively; Figure 2B). Note that ΔD2R was lower than ΔD1R, even though trial durations were shorter for D2R-KO than D2R-dWT mice (a favorable condition for a positive value of ΔD2R).

### Performance in the TAB task

**Figure 3:**
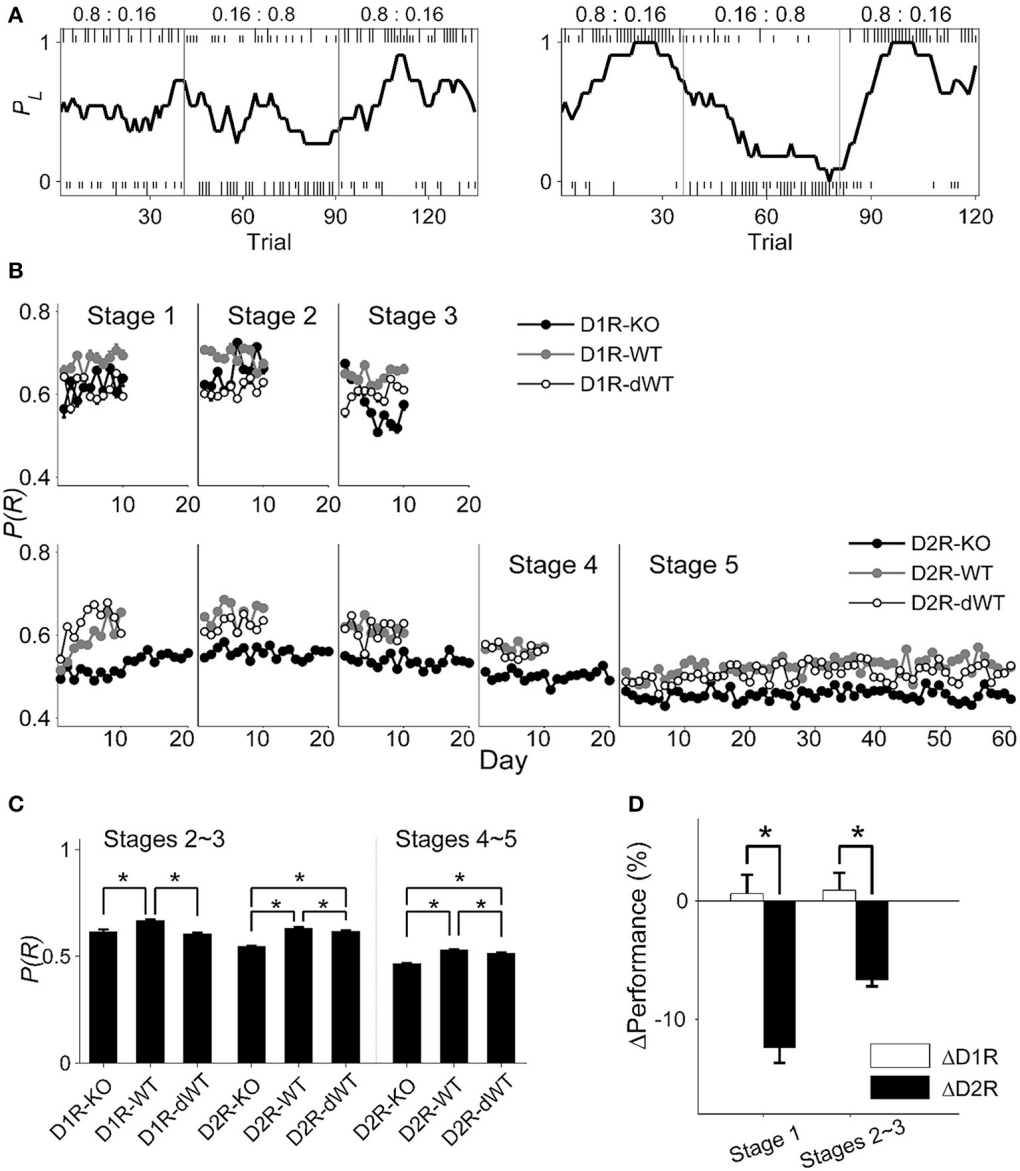
**Behavioral performance in the dynamic TAB task. (A)** Examples of choice behavior (chosen from stage 3; left, D2R-KO; right, D2R-dWT) in the TAB task. Tick marks indicate trial-by-trial choices of the animal (top, left choice; bottom, right choice; long, rewarded; short, unrewarded) and vertical lines denote block transitions. Numbers indicate reward probabilities in each block. The line shows the probability of choosing the left goal (*P*_*L*_) in moving average of ten trials. **(B)** The proportion of rewarded choices [P(R)] is shown for each daily session for each experimental group. **(C)** Behavioral performance data (mean ± s.e.m. across animals) were pooled across days for stages 2–3 and 4–5. **(D)** Relative performance (mean ± s.e.m. across daily sessions) is shown for stages 1 and 2–3. Asterisks, significant difference [**(C)**, One-Way ANOVA followed by Bonferroni *post-hoc* tests; **(D)**, *t*-test, *p* < 0.05)].

The animal's performance was assessed by the proportion of rewarded choices [P(R)] (session examples are shown in Figure 3A), but similar results were obtained when it was assessed by the proportion of choosing the higher arming probability goal in each block (data not shown). In stage 1, performances of D1R-KO and D1R-dWT mice were not significantly different from each other, but significantly lower than that of D1R-WT mice (main effect of group, *p* = 0.002; D1R-KO vs. D1R-WT, *p* = 0.033; D1R-KO vs. D1R-dWT, *p* = 1.000; D1R-WT vs. D1R-dWT, *p* = 0.003). By contrast, performance of D2R-KO was significantly lower than those of D2R-WT and D2R-dWT mice (data during the initial 10 d were analyzed; main effect of animal group, *p* = 1.0 × 10^−7^; D2R-KO vs. D2R-WT, *p* = 1.0 × 10^−7^; D2R-KO vs. D2R-dWT, *p* = 0.010; Figure 3B).

Daily performance of the animals stayed more or less stable in the subsequent stages (Figure 3B). We therefore collapsed behavioral data of stages 2–3 and those of stages 4–5 and analyzed them together. In stages 2–3, performances of D1R-KO and D1R-dWT mice were similar, but significantly lower than that of D1R-WT mice (One-Way ANOVA, *p* = 1.0 × 10^−7^; Bonferroni *post-hoc* test, D1R-KO vs. D1R-WT, *p* = 1.0 × 10^−7^; D1R-KO vs. D1R-dWT, *p* = 0.963; D1R-WT vs. D1R-dWT. *p* = 1.0 × 10^−7^) as in stage 1, suggesting that impaired performance of D1R-KO relative to D1R-WT animals was because of longer trial durations. On the other hand, D2R-KO mice showed significantly lower performance than the other D2R animal groups in stages 2–3 (*p* = 1.0 × 10^−7^; D2R-KO vs. D2R-WT, *p* = 1.0 × 10^−7^; D2R-KO vs. D2R-dWT, *p* = 1.0 × 10^−7^) as well as in stages 4–5 (*p* = 1.0 × 10^−7^; D2R-KO vs. D2R-WT, *p* = 1.0 × 10^−7^; D2R-KO vs. D2R-dWT, *p* = 1.0 × 10^−7^; Figure 3C). Regression analysis considering trial duration also indicated no significant effect of D1R-KO in stages 2–3 (*t*-test, *p* = 0.434), but significant effects of D2R-KO in stages 2–3 (*p* = 3.9 × 10^−48^) and 4–5 (*p* = 1.9 × 10^−54^). In addition, relative performance of D2R-KO mice (ΔD2R) was significantly lower compared to that of D1R-KO mice (ΔD1R) in stage 1 (initial 10 days, *t*-test, *p* = 9.9 × 10^−6^) as well as stages 2–3 (*p* = 9.6 × 10^−5^; Figure 3D).

### Effects of past choices and rewards

**Figure 4:**
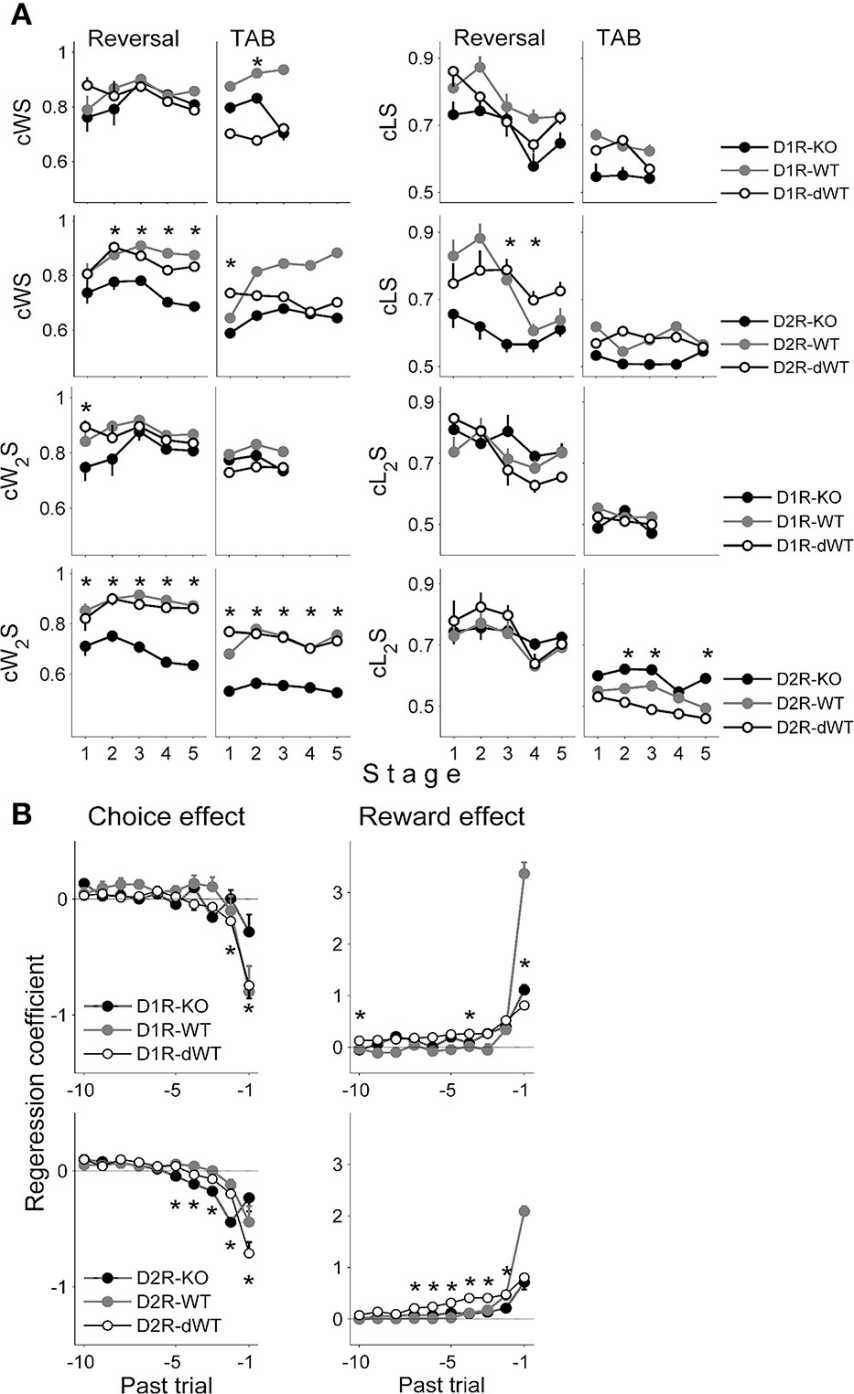
**Effects of past choices and rewards on the animal's choice. (A)** Proportions of cWS (repeating rewarded choice in the previous trial), cLS (switching from unrewarded choice in the previous trial), cW_2_S (repeating rewarded choice two trials before) and cL_2_S (switching from unrewarded choice two trials before) are shown for each behavioral stage. Asterisks, significant difference between KO and dWT mice (Two-Way repeated measure ANOVA followed by Bonferroni *post-hoc* tests, *p* < 0.05). **(B)** Results of a logistic regression analysis. Effects of past choices and rewards (up to 10 trials) on the animal's choice in the current trial were estimated by applying a logistic regression analysis to the behavioral data obtained during the TAB task (D1R, stages 1–3; D2R, stages 1–5). A positive coefficient for the choice effect indicates the animal's tendency to repeat the same choice, and a positive coefficient for the reward effect indicates the animal's tendency to repeat the rewarded choice. Error bars, 95% confidence intervals. Asterisks, significant difference between KO and dWT mice (One-Way ANOVA followed by Bonferroni *post-hoc* tests, *p* < 0.05).

In order to examine how the animal's current choice was influenced by the animal's choice and its outcome in the previous trial, we assessed the proportions of repeating rewarded choice (combined win-stay or cWS) and switching from unrewarded choice (combined lose-switch or cLS). Note that we call these measures as cWS and cLS to denote combined effects of potential multiple underlying processes and to distinguish them from pure WS and LS that are independent of the other components of the model (such as the RL term; see Materials and Methods). We additionally examined effects of the animal's choice and its outcome two trials before on the current choice by assessing proportions of repeating the choice that was rewarded two trials before (cW_2_S) and switching from the choice that was unrewarded two trials before (cL_2_S). In the reversal task, cWS and cLS tended to be lower in D2R-KO than D2R-dWT mice (Two-Way repeated measure ANOVA followed by Bonferroni *post-hoc* tests, cWS, stages 2–5, *p*-values < 0.05, cLS, stage 3, *p* = 0.009; stage 4, *p* = 0.047), but similar between D1R-KO and D1R-dWT mice (cWS, *p*-values > 0.1; cLS, *p*-values > 0.20; Figure 4A), which is consistent with impaired performance of D2R-KO mice in the reversal task. In the TAB task, no significant difference was found in these measures between D2R-KO and D2R-dWT mice except cWS in stage 1 (first 10 trials of stages 1–4 were used for D2R-KO mice for statistical comparisons with the other mice; cWS, stage 1, *p* < 0.001; stages 2–5, *p* > 0.18; cLS, all stages, *p*-value > 0.09). On the other hand, cW_2_S was significantly lower (all training stages, *p*-values < 0.002) and cL_2_S was significantly higher (stages 2, 3 and 5, *p*-values < 0.002) in D2R-KO than D2R-dWT mice. No significant difference was observed for these measures between D1R-KO and D1R-dWT mice (stages 1–3, cW_2_S, *p*-values > 0.5; cL_2_S, *p*-values > 0.75; Figure 4A). Thus, in the TAB task, D2R-KO animals were different from D2R-dWT mice in incorporating distant reward history (reward at *t-2* trial) in deciding which goal to choose.

We also ran a logistic regression analysis to examine further how the animal's choices were influenced by the history of past choices and their outcomes during the TAB task. All animals tended to alternate their choices (choice effect) whereas repeat the choice that was rewarded in recent trials (reward effect) as previously described for rats (Huh et al., 2009; Kim et al., 2009) and monkeys (Lau and Glimcher, 2005). However, effects of past choices and rewards were different across WT, dWT, and KO animal groups for both D1R and D2R (Figure 4B), and the following characteristics are worth noting. First, reward effect of the previous trial (*t-1*) in WT animals was markedly different from those of dWT and KO animals for both D1R and D2R, indicating a strong effect of trial duration on this measure. Second, effects of past choices and rewards were different between KO and dWT groups for both D1R and D2R (One-Way ANOVA followed by Bonferroni *post-hoc* tests), indicating that not only D2R-KO, but also D1R-KO altered the way past choices and rewards influenced the animal's choices. Thus, although D1R-KO mice showed relatively intact performance in the TAB task, the pattern of their choices was different from those of the other animal groups. Third, past choice effect was not a monotonic function for D2R-KO mice. The magnitude of past choice effect increased between *t-1* and *t-2* trials and then gradually declined for more distant trials. Although results of a simple regression analysis are limited in providing useful information on underlying neural processes, this pattern raises a possibility that effects of past choices and rewards are mediated by multiple underlying processes.

### Modeling

The above analysis results suggest altered choice behavior of D1R-KO and D2R-KO mice from their respective control mice (D1R-dWT and D2R-dWT, respectively). However, they are limited in revealing underlying psychological/neural processes because externally observed measures might be outcomes of combined effects of multiple underlying processes. For example, win-stay can be influenced by an RL-like process, wherein actions are selected according to values that are computed based the history of past choices and rewards, as well as by a simple win-stay-lose-switch strategy irrespective of values. We therefore performed a model-based analysis to obtain insights on psychological/neural processes underlying the animal's choice behavior. We have shown previously that rat's choice behavior in a dynamic TAB task similar to the one used in the present study is well explained by a simple RL model (Huh et al., 2009). However, the non-monotonic influence of past choices in D2R-KO mice (Figure 4B) suggests existence of multiple processes mediating effects of past choices and rewards. Also, previous studies added an additional RL process (Beeler et al., 2010), a perseveration factor (Rutledge et al., 2009) or a win-stay-lose-switch strategy (Worthy and Maddox, 2014) to an RL model to account for humans' or mice's choice behavior. In addition, an uncertainty-based exploration term was added to an RL model to account for choice behavior of human subjects carrying different alleles for genes controlling dopamine functions (Frank et al., 2009). We therefore examined several different versions of a hybrid model, and found that a model consisting of a win-stay-lose-switch strategy (irrespective of value), a simple RL component (which updates value in a recursive manner) and uncertainty-based exploration well explained the animal's choice behavior in the TAB task. Specifically, the model containing separate processes for win-stay (WS, repeating the rewarded choice in the previous trial irrespective of value), lose-switch (LS, switching from the unrewarded choice in the previous trial irrespective of value), value learning from positive outcome, value learning from negative outcome and uncertainty-based exploration along with choice bias outperformed all other reduced models as assessed by Akaike's information criterion (AIC) and Bayesian information criterion (BIC) (Burnham and Anderson, 2002) (Table 1).

**Table 1:** **Results of model comparison**.

	**[RL, Bias, Stay]**	**[RL, Bias, Stay, UE]**	**[RL, Bias, WS, LS]**	**[RL, Bias, WS, LS, UE]**
AIC	0	1	2	38
BIC	2	3	4	32

*Each number indicates the number of animals whose choice behavior in the TAB task (total n = 41) was best explained by a given model in terms of AIC or BIC. The full model explained choice behavior best in the majority of animals. RL, reinforcement learning term; Bias, bias to choose one particular goal; UE, uncertainty-based exploration; WS, win-stay; LS, lose-switch; Stay, perseveration factor (tendency to stay regardless of choice outcome)*.

**Figure 5:**
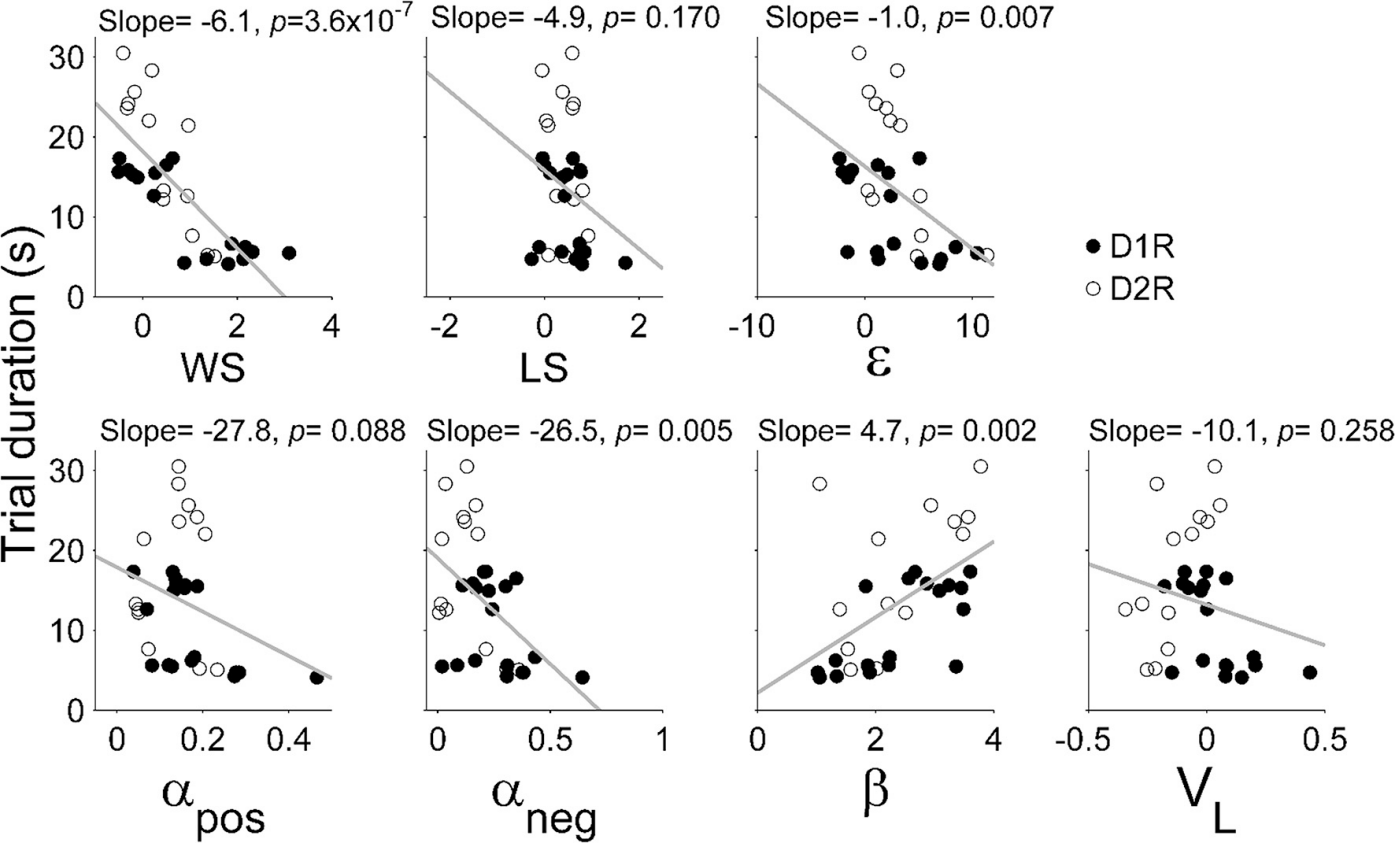
**Correlations between trial duration and model parameters**. WS (*S*_*reward*_), win-stay; LS (*S*_0_), lose-switch; ε, coefficient for uncertainty-based exploration; α_*pos*_, learning rate for positive outcome; α_*neg*_, learning rate for negative outcome; β, inverse temperature indicating randomness in action selection; *V*_*L*_, choice bias toward the left goal. All animals tested in the TAB task were included in the analysis.

**Figure 6:**
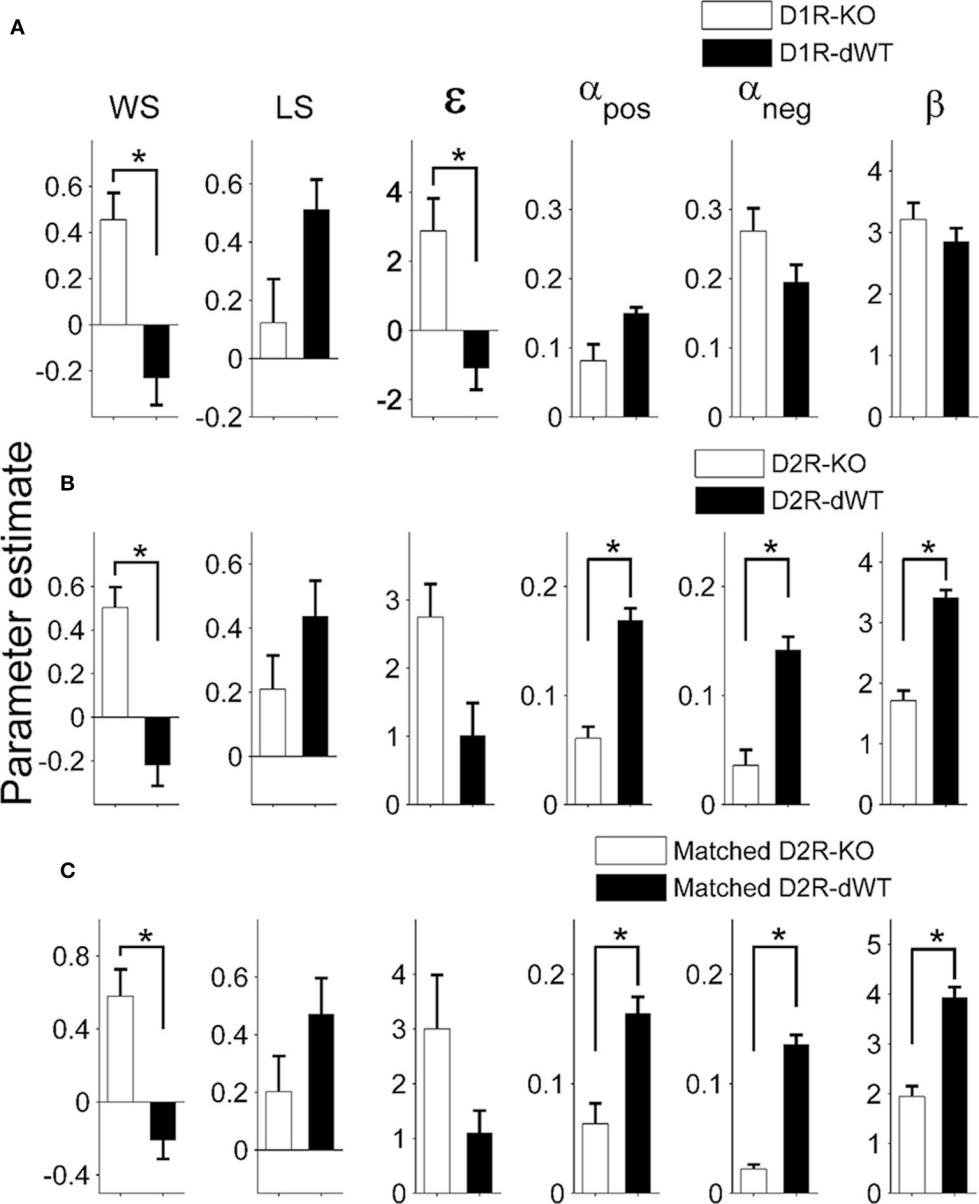
**Model parameters**. Shown are coefficients for the explanatory variables of the full model applied to the behavioral data obtained during the TAB task (D1R, stages 1–3; D2R, stages 1–5; mean ± s.e.m. across animals). Asterisks, significant differences (Wilcoxon rank sum test, *p* < 0.05). **(A,B)** Values of model parameters obtained from D1R-KO and D1R-dWT mice **(A)**, and those obtained from D2R-KO and D2R-dWT mice **(B)** are shown. **(C)** Values of model parameters for D2R-KO and D2R-dWT mice were estimated after matching trial durations between D2R-WT and D2R-KO mice.

Results of the logistic regression analysis (Figure 4B) indicated that trial duration strongly affected the influence of the previous reward on the animal's subsequent choice in the TAB task. As can be expected from this, trial duration was significantly correlated with the majority of model parameters (Figure 5). Based on this observation, we focused on comparing model parameters between KO and dWT animals. For D1R, WS (*S*_*reward*_) and uncertainty-based exploration (ε) were significantly higher for KO than dWT animals (Wilcoxon rank sum test, *p* = 0.048 for both parameters; Figure 6A). For D2R, WS was significantly higher (*p* = 0.001), and value learning from positive outcome (α_*pos*_), value learning from negative outcome (α_*neg*_) and inverse temperature controlling randomness in action selection (β) were significantly lower (*p* = 0.001, 0.007, and 0.001, respectively) for KO than dWT animals (Figure 6B). To test the possibility that differences in model parameters between D2R-KO and D2R-dWT mice were because of different trial durations between these animal groups, we repeated the same analysis after matching trial durations of D2R-KO and D2R-dWT animal groups by excluding long (D2R-dWT, 244 out of 500) and short (D2R-KO, 244 out of 700) behavioral sessions of the TAB task (resulting trial durations, D2R-dWT, 21.3 ± 0.4 s; D2R-KO, 21.3 ± 0.5 s; *t*-test, *p* = 0.980). The analysis yielded similar results (Figure 6C). This result might appear inconsistent with significant correlations between trial duration and model parameters (Figure 5). However, the difference in trial duration between D2R-KO and D2R-dWT mice was relatively small compared to that between D2R-KO and D2R-WT mice and, more importantly, a given amount of change in trial duration would have only a weak effect when the original trial duration is relatively long because reward effect presumably decays over time according to an exponential or hyperbolic function (Kalenscher and Pennartz, 2008). Consistent with these accounts, no model parameter except one (α_*pos*_; even in this case a positive, rather than negative, correlation was found suggesting a possibility of spurious correlation) showed a significant correlation with trial duration when the subjects with mean trial durations <10 s were excluded (data not shown).

**Figure 7:**
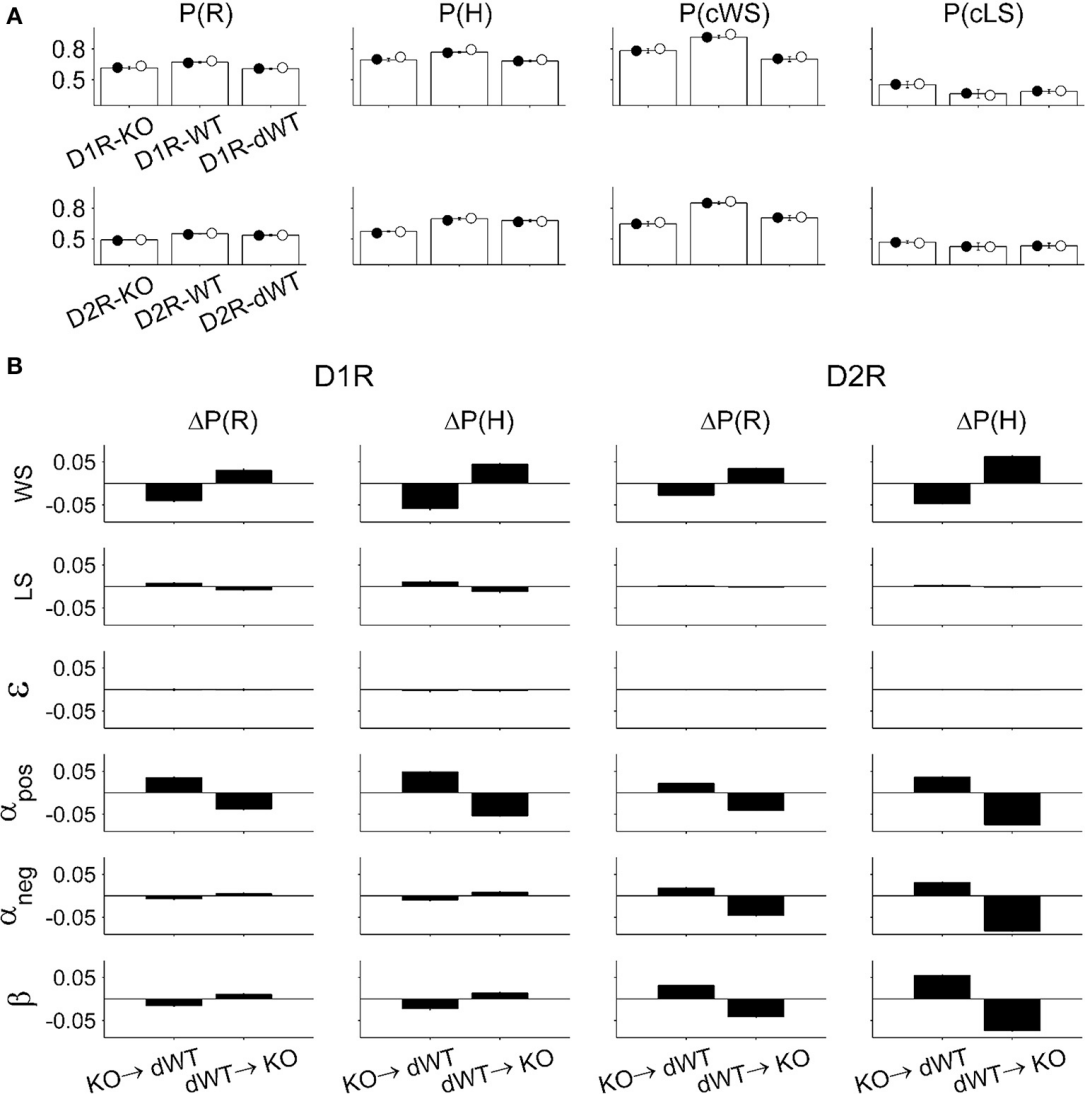
**Model prediction. (A)** Animal's choices were simulated with the full model. P(R), the proportion of rewarded choices; P(H), the proportion of choosing the higher arming-probability goal in each block; P(cWS), the proportion of repeating rewarded choice in the previous trial; P(cLS), the proportion of switching from unrewarded choice in the previous trial. Open bars, actual choice data (mean ± s.e.m. across animals); filled circles, choices were predicted using model parameters estimated for individual animals (mean across animals); open circles, choices were predicted using model parameters averaged across animals in each experimental group (mean across animals). **(B)** Effect of changing a model parameter. KO→dWT, effect of replacing a model parameter of KO mice with that of dWT mice. The graphs indicate altered P(R) and P(H) [ΔP(R) and ΔP(H), respectively] relative to those of KO mice. dWT→KO, effect of replacing a model parameter of dWT mice with that of KO mice. The graphs show altered P(R) and P(H) relative to those of dWT mice.

Choices predicted by the full model using parameters obtained from the animals matched actual choices of the animals during the TAB task quite well. The proportion of rewarded choices [P(R)], the proportion of choosing the higher arming-probability goal [P(H)], the proportion of repeating rewarded choice in the previous trial [P(cWS)], and the proportion of switching from unrewarded choice in the previous trial [P(cLS)] were similar between the actual and predicted data (Figure 7A). To gain insights on how choice behavior of KO mice was influenced by a particular component of the model, we examined effects of replacing a model parameter on behavioral performance of the model. When the value of a particular model parameter of D2R-KO mice was replaced with that of D2R-dWT mice, the performance [P(R)] was enhanced for α_*pos*_, α_*neg*_, and β, but decreased for WS compared with the performance of D2R-KO mice. Conversely, when a model parameter value of D2R-dWT mice was replaced with that of D2R-KO mice, the performance decreased for α_*pos*_, α_*neg*_, and β, but increased for WS compared with the performance of D2R-dWT mice (Figure 7B). These results indicate that altered α and β contributed to impaired choice behavior of D2R-KO mice in the TAB task, which was alleviated by altered WS. For D1R-KO mice, increased WS enhanced the animal's performance, which was offset by decreased α_*pos*_ (Figure 7B), although α_*pos*_ was not significantly different between D1R-KO and D1R-dWT mice (*p* = 0.095). Replacing the value of ε had little effect on the performance of D1R or D2R animals (Figure 7B).

## Discussion

We examined choice behavior of D1R-KO and D2R-KO mice while varying stability and certainty of action-reward contingency. Although all animals learned to choose correct target in the simple instrumental learning task, performance of D2R-KO mice was impaired as stability and certainty of action-reward contingency decreased, whereas performance deficits of D1R-KO were relatively small. A model-based analysis indicated increased win-stay tendency, but impaired value updating and decreased value-dependent action selection in D2R-KO mice, which was detrimental to making optimal choices in the TAB task. These results indicate importance of D2R in learning from the history of past choices and their outcomes for rapid adjustment of choice behavior in a dynamic and uncertain environment.

### Role of D2R in rapid adjustment of choice behavior

It has been proposed that dopamine is involved in stimulus-reward, but not response-reward association (Berridge, 2007; Flagel et al., 2011). However, our results indicate requirement of dopamine in response-reward association when action-reward contingency is unstable. All animals learned to choose the correct goal >80% during the initial training and after a single episode of reversal. However, performance of D2R-KO mice was dramatically impaired in the subsequent phases of reversal training, which is consistent with previous studies showing involvement of D2R in reversal learning (Lee et al., 2007; Pizzagalli et al., 2008; Boulougouris et al., 2009; Cools et al., 2009; De Steno and Schmauss, 2009; Jocham et al., 2009; Herold, 2010; Groman et al., 2011; van der Shaaf et al., 2013), although involvement D1R in reversal learning has also been reported (Diekamp et al., 2000; Calaminus and Hauber, 2007). It is notable that D2R-KO mice were profoundly impaired when reward uncertainty was added to the task (i.e., TAB task) even with prolonged training, which is in line with impaired performance of Parkinson's disease (PD) patients in probabilistic learning tasks (Knowlton et al., 1996; Shohamy et al., 2004). These findings suggest that dopamine, largely through D2R, plays an essential role in rapid adjustment of choice behavior in a dynamic environment, whereas gradual adjustment of behavior in a stable environment does not require intact D2R.

It is unclear why the absence of D2R led to more severe performance deficits compared to the absence of D1R in a dynamic and uncertain environment. Anatomical distributions of D1R and D2R are different across brain structures (e.g., relatively high levels of D1R in the cerebral cortex; Hurley and Jenner, 2006), within a brain structure (e.g., D1R and D2R mRNAs are expressed primarily in layers 6 and 4–5, respectively, in rat neocortex; Weiner et al., 1991), and across cell types (e.g., D1R and D2R expressions in striatal neurons projecting to substantia nigra pars reticulata and globus pallidus, respectively; Missale et al., 1998; Kreitzer and Malenka, 2008). In addition, physiological effects of D1R and D2R activation are different (e.g., D2R, but not D1R, functions as a presynaptic autoreceptor regulating dopamine release; Hurley and Jenner, 2006; Romanelli et al., 2009). Any of these factors can be responsible for different choice behavior of D1R-KO and D2R-KO mice, which remains to be determined.

### Learning from RPE

Our model-based analysis indicated that D2R-KO mice were impaired in updating value based on RPE, which supports the proposed role of dopamine in RPE-based learning as postulated by the RL theory. Our analysis also indicated D2R involvement in learning from both positive and negative RPE. It has been controversial whether dopamine is involved in learning from only positive RPE (Morris et al., 2004; Bayer and Glimcher, 2005; Pessiglione et al., 2006; Rutledge et al., 2009; Fiorillo, 2013) or both positive and negative RPE (Frank et al., 2004, 2007, 2009; Bayer et al., 2007; Klein et al., 2007; Hart et al., 2014), and roles of D1R vs. D2R in RPE-based learning are unclear. In humans, variations of *DARPP-32* and *D2RDD* genes, which are related to D1R and D2R functions, respectively, were correlated with learning from positive and negative RPE, respectively (Frank et al., 2007; Klein et al., 2007). Subsequent studies in rodents employing specific manipulations of striatal D1R and D2R have yielded consistent results (Hikida et al., 2010, 2013; Kravitz et al., 2012; Tai et al., 2012; Danjo et al., 2014). However, in monkeys, striatal D2R availability was correlated with learning from positive, but not negative, feedback (Groman et al., 2011; but see Piray, 2011). The reason for inconsistent findings across studies is currently unclear. Such factors as different anatomical distributions of D1R and D2R across different animal species (Mandeville et al., 2011), global vs. focal manipulations of dopamine receptors, chronic vs. transient manipulations of dopamine receptors, and different degrees of dopamine receptor manipulations (e.g., relatively small quantitative variations in dopamine receptor functions caused by genetic variations in humans vs. complete knock-out of dopamine receptors in the present study) might have contributed to inconsistent results. Despite such inconsistency, results from these studies are all consistent in that D2R is involved in RPE-based learning.

### Value-dependent action selection

Decreased value-dependent action selection (decreased β) was another important factor for impaired performance of D2R-KO mice in the TAB task. A previous modeling study has suggested that increased tonic dopamine in the basal ganglia might decrease β via D1R (Humphries et al., 2012). Our results show, however, that D2R, rather than D1R, is important for controlling β. Another study has found decreased β in dopamine transporter-KO mice (hyperdopaminergic mice) (Beeler et al., 2010). The relationship between this finding and ours is unclear. A common process might have been affected in the same direction by D2R-KO and dopamine transporter KO. For example, absence of D2R autoreceptors (Romanelli et al., 2009) might lead to enhanced dopamine release, which in turn causes decreased value-dependent action selection. Although additional studies are needed to clarify this issue, both studies provide evidence for the involvement of dopamine in controlling value-dependent action selection, and our study indicates importance of D2R, rather than D1R, for this process.

### Win-stay

Both D1R-KO and D2R-KO animals showed increased WS compared to their delay-matched control animals. These results are different from, but consistent with the previous findings that PD patients off-medication (Rutledge et al., 2009) and rats with dorsal striatal lesions (Skelin et al., 2014) tended to repeat the same choice, raising the possibility that D1R-KO and D2R-KO effects on WS found in the present study might be mediated by dorsal striatum. Enhanced WS in D2R-KO mice alleviated performance deficit in the TAB task. Increased tendency to repeat the choice rewarded in the previous trial would facilitate performance in many behavioral settings, particularly in a simple instrumental learning task. However, such a simple strategy without considering values computed based on the history of past rewards would be suboptimal in a dynamic and uncertain environment, such as during a TAB task.

### Uncertainty-based exploration

In humans, a gene known to primarily control prefrontal dopamine function (*catechol-O-methyltransferase*) was associated with uncertainty-based exploration. Specifically, the *val* allele (low dopamine function) was associated with reduced exploration compared to the *met* allele (high dopamine function) (Frank et al., 2009). We found that D1R-KO increased uncertainty-based exploration compared to delay-matched control mice. The two studies are consistent in that dopamine is related to uncertainty-based exploration and that D1R is more abundant than D2R in the prefrontal cortex (Seamans and Yang, 2004; Hurley and Jenner, 2006). However, they are inconsistent in that low dopamine function was associated with low (the *val* allele in humans) or high (D1R-KO in mice) exploration. The relationship between dopamine function and uncertainty-based exploration might be a non-monotonic function. Alternatively, considering that dopamine can act at low and high concentrations on D1R and D2R, respectively, to exert opposing physiological actions in the prefrontal cortex (Seamans and Yang, 2004), both elevated dopamine and absence of D1R might end up with similar functional consequences via relatively enhanced D2R functions, which remains to be explored. Changes in uncertainty-based exploration had little effect on the animal's choice behavior in our dynamic TAB task. However, choice behavior of D1R-KO mice may deviate substantially from that of control animals when uncertainty-based exploration is a critical factor for maximizing rewards.

### Multiple roles of dopamine in mediating reward effects

RL models have been successful in accounting for choice behavior of humans and animals (Dayan and Niv, 2008; Niv and Montague, 2009; Lee et al., 2012). The results of our model comparison showed, however, that a simple RL model alone is insufficient to describe choice behavior of mice in the TAB task. Additional components, namely a win-stay-lose-switch strategy and uncertainty-based exploration, were necessary to better describe mice's choice behavior. Previous studies have shown that adding a win-stay-lose-switch strategy (Worthy and Maddox, 2014), a perseveration factor (Rutledge et al., 2009) or an additional RL term with a short time constant (Beeler et al., 2010) in addition to an RL model better accounted for humans' or mice's choice behavior. Thus, multiple effects of reward might be a general characteristic across different animal species. Our modeling results indicate multiple roles of dopamine in mediating diverse reward effects, which is consistent with previous findings in humans (Frank et al., 2009). Specifically, D1R was involved in controlling WS and uncertainty-based exploration, and D2R was involved in controlling WS, value updating and value-dependent action selection. These results suggest that dopamine is involved in not only learning from RPE, but also another component of RL, namely controlling value-dependent action selection, as well as other aspects of reward processing that are not described by a simple RL model.

### Limitations of the study

Although our study provides new insights on functional roles of D1R and D2R, there remain outstanding issues that need to be addressed in future studies. First, we cannot rule out the possibility that developmental changes or compensation mechanisms associated with dopamine receptor KO largely contributed to the observed behavioral changes. Second, specific brain areas and the mode of dopaminergic neuronal activity (tonic vs. phasic) mediating the proposed functions of D2R are unknown. Previous studies have shown that distinct neural signals related to value-based decision making are observed in various dopaminoceptive areas of the brain (Lee et al., 2012), raising the possibility that effects of dopamine manipulation in each of these brain structures might induce distinct effects on choice behavior. Future investigations using region-specific (such as targeting prefrontal D1R for its involvement in uncertainty-based exploration and striatal D2R for its role in value-dependent action selection) and time-controlled (i.e., adult stage-specific) inactivation of dopamine receptors (such as inducible KO, optogenetic manipulation and siRNA-based strategy) along with activity mode-specific manipulation of dopamine neurons (Zweifel et al., 2009; Schiemann et al., 2012) would be necessary to address these concerns.

## Author contributions

Shinae Kwak and Min W. Jung designed the study; Ji-Seon Seo, Jung-Eun Lee, and Pyung-Lim Han produced animal subjects; Shinae Kwak collected behavioral data; Shinae Kwak, Namjung Huh, and Min W. Jung analyzed the data; Namjung Huh conducted modeling; Min W. Jung wrote the manuscript with inputs from all other authors.

### Conflict of interest statement

The authors declare that the research was conducted in the absence of any commercial or financial relationships that could be construed as a potential conflict of interest.
